# Quasi-static pipeline in electroconvulsive therapy computational modeling

**DOI:** 10.1016/j.brs.2023.03.007

**Published:** 2023-03-16

**Authors:** Gozde Unal, Cynthia Poon, Mohamad FallahRad, Myesha Thahsin, Miklos Argyelan, Marom Bikson

**Affiliations:** a Department of Biomedical Engineering, The City College of New York, CUNY, New York, NY, USA; b Center for Neurosciences, The Feinstein Institute for Medical Research, North Shore- Long Island Jewish Health System, Manhasset, NY, 11030, USA

## Abstract

**Background:**

Computational models of current flow during Electroconvulsive Therapy (ECT) rely on the quasi-static assumption, yet tissue impedance during ECT may be frequency specific and change adaptively to local electric field intensity.

**Objectives:**

We systematically consider the application of the quasi-static pipeline to ECT under conditions where 1) static impedance is measured before ECT and 2) during ECT when dynamic impedance is measured. We propose an update to ECT modeling accounting for frequency-dependent impedance.

**Methods:**

The frequency content on an ECT device output is analyzed. The ECT electrode-body impedance under low-current conditions is measured with an impedance analyzer. A framework for ECT modeling under quasi-static conditions based on a single device-specific frequency (e.g., 1 kHz) is proposed.

**Results:**

Impedance using ECT electrodes under low-current is frequency dependent and subject specific, and can be approximated at >100 Hz with a subject-specific lumped parameter circuit model but at <100 Hz increased non-linearly. The ECT device uses a 2 μA 800 Hz test signal and reports a static impedance that approximate 1 kHz impedance. Combined with prior evidence suggesting that conductivity does not vary significantly across ECT output frequencies at high-currents (800—900 mA), we update the adaptive pipeline for ECT modeling centered at 1 kHz frequency. Based on individual MRI and adaptive skin properties, models match static impedance (at 2 μA) and dynamic impedance (at 900 mA) of four ECT subjects.

**Conclusions:**

By considering ECT modeling at a single representative frequency, ECT adaptive and non-adaptive modeling can be rationalized under a quasi-static pipeline.

## 1. Introduction

### 1.1. Questions about the quasi-static assumption in ECT

The quasi-static assumption is ubiquitous in modeling of current flow through the body during electrical stimulation [1-4]. Under the quasi-static assumption, tissue current flow is linear with applied stimulation intensity and independent of frequency. This follows from tissues being assigned fixed conductivities (independent of frequency or intensity) and tissue permittivity (capacitance) assumed negligible, including at the electrodes/tissue interfaces (cf [5]). The quasi-static assumption is common in simulations of Electroconvulsive Therapy (ECT) [6-8]. Practically, such simplifications lend themselves well to the finite element method (FEM) approaches, including models representing detailed anatomy [8,9].

Based on the resulting current flow (electric fields) across tissue including the brain, either non-linear functions (e.g., action potentials, connectivity; [10,11]) or the quasi-uniform assumption [12-14] are then applied to predict changes in brain function. For example, in ECT modeling the quasi-uniform assumption can be applied with a threshold. Nevertheless, current flow itself is conventionally assumed insensitive to stimulation intensity or frequency.

Yet, for skin impedance, the dependence on frequency along with intensity has been documented for decades [15,16], leading to non-linear lumped parameter models (e.g. with layer/structure-specific complex impedance [17]). This is further complicated by the changing state of skin (e.g., with environment, time) and the interaction with the electrode/electrode-skin interface – these factors can be avoided in experimental studies characterizing skin impedance but are central in clinical measurements intended to be sensitive to proper electrode set-up. Models of transcranial electrical stimulation maintain skin at a fixed simple conductivity.

We previously suggested that features specific to ECT dose, namely very high intensity (compared to low-intensity tES such as tDCS/tACS) and application of current through the skin (compared to invasive approaches or magnetic stimulation), creates special conditions of very high electric fields in the skin, and that these in turn change skin conductivity. This is clear from clinical measurements of high “static impedance” (measured with bespoke low-intensity test currents prior to ECT) compared to a low “dynamic impedance” (measured during ECT high currents). To model this with FEM, we developed adaptive models of ECT [18] whereby superficial skin conductivity increases with local electric field. This modeling pipeline included both individual anatomy and individual skin properties to match clinical impedance data. Notwithstanding the need to iteratively search for result that satisfy adaptive constraints, the adaptive models physics remained quasi-static. However, the nuanced nature of impedance during ECT also raises a question about the further role of frequency in skin impedance.

### 1.2. A novel approach for ECT modeling based on one representative frequency

To fully simulate non-linear skin (and skin/electrode interface) properties is both computationally intractable and muddled by lack of skin impedance data for ECT waveforms. On the other hand, how can complex skin impedance be ignored if it affects impedances reported by ECT devices and current flow patterns? Here we resolve these issues by developing an ECT modeling pipeline that is tractable, requires minimal parameterization, adaptive [18], quasi-static, and matches subject-specific measurements without ignoring complex skin impedance. This novel approach is based around modeling conductivity at a device-specific single frequency. The explanation below is based on selecting 1 kHz as this frequency.

Our objectives are limited to the study of the physical properties of ECT, including subject specific models, and the value of parametrizing static impedance and dynamic impedance. At low current intensities used for static impedance, we show there is a high degree of frequency sensitivity. We consider that the static impedance as reported a ECT device can be modeled by assigning tissue conductivities for one frequency – in this case 1 kHz. At high current intensities, we suggest tissue conductivity is frequency insensitive between ~0.01 and 2 kHz, a range that spans the frequency content of the ECT waveform and includes the representative 1 kHz frequency. Thus, using two different rationales, considering tissue conductivity at 1 kHz addresses the static impedance and ECT dynamic impedance cases.

For adaptive ECT FEM models [18], there are three tissue cases to consider. 1) For each non-skin compartments (eg. skull, CSF/meninges, gray and white matter), we assign fixed and subject-invariant conductivities. These conductivities can be considered at 1 kHz, or rather to be frequency independent so applicable to all relevant frequencies (~0.01–2 kHz) [19]. 2) Deep-skin is assigned a fixed subject-specific conductivity, which can similarly be considered representative at 1 kHz or frequency-independent. 3) Superficial skin is adaptive, decreasing as local electric field increases. At low-current, this conductivity is specific to 1 kHz, while at high intensities this conductivity represents ~0.01–2 kHz (as conductivity does not vary in this range) which spans 1 kHz. The transfer function (see Methods) between superficial skin electric field and conductivity is thus for 1 kHz.

For all tissues, permittivity (capacitance) is assumed insignificant, and any frequency dependent conductivity accommodated through the representative-frequency assumptions. The electrodes are fixed conductivity. The impact of the electrode-skin interface (e.g., skin preparation; [18]) is captured by underlying superficial-skin properties. In sum, with all compartments modeled by conductivity at 1 kHz (or assumed frequency-independent) the quasi-static pipeline can be applied to predict ECT current flow and impedances.

### 1.3. Implications for explaining ECT devices

The validity and application of modeling conductivities at a representative-frequency (in the device specific case of 1 kHz) as supported with physical measurements of impedance is the basis of this paper. Regarding expediency, this approach does not require technical changes to either standard or adaptive ECT modeling workflows [18] except a quasi-static pipeline is not applicable by considering conductivities at 1 kHz. In regards to skin biophysics, we avoid decades-long debate on the (sub)cellular mechanisms [17,20] governing the skin’s complex impedance. We only anticipate our representative-frequency approach models local tissue conductivity as it impacts relevant ECT physics.

Are ECT devices designed to report a static impedance approximating the impedance at a single frequency (e.g. 1 kHz) - so that any differences between devices [18] reflect different test frequencies? No. Rather, ECT devices employ heuristic circuits, test signals, and processing to report a value of ‘static impedance’. Static impedance is intended only to alert the operator to poor electrode set-up and the possibility of a dynamic impedance above the ECT device voltage compliance. Only in this last sense is correlation between static and dynamic impedance currently important in practice (see Ref. [18]).

Are ECT devices designed to report a dynamic impedance approximating the impedance at ~1 kHz? They are not. Rather, ECT devices report a value of ‘dynamic impedance’ determined from the required peak voltage to generate ECT current pulses (~800–900 mA). We anticipate the impedance at 1 kHz under high intensity approximates the reported dynamic impedance by the ECT device.

## 2. Methods

### 2.1. Thymatron device waveform analysis during static and dynamic impedance measurements

We used resistors representing typical static (2200 kΩ) and dynamic (300 Ω) impedances, and measured the device output voltage. The current output over time was calculated by dividing the voltage by the resistance of the resistor for each impedance measurement. A Fast Fourier Transform was computed using the Matlab fft() function [21]. The two sided amplitude spectrum of the FFT was converted to a single sided amplitude spectrum. We also simulated idealized ECT outputs (20 Hz, alternating pulse polarity) with varied pulse durations (0.25 m s, 0.5 m s, 1 m s) and similarly computed the frequency spectrum.

### 2.2. Experiment series 1: Static impedance vs. impedance spectrum experiments on healthy subjects

We conducted a study to evaluate the impedance using adhesive ECT electrodes at low currents in healthy subjects (n = 10). The study was conducted in accordance with protocols and procedures approved by the Institutional Review Board of the City University of New York. All experiments were made using disposable, semiadherent electrodes (Thymapads, Somatics, LLC, Venice, FI). The electrode preparation techniques included cleaning skin with saline and Pretac application (Pharmaceutical Innovations, Newark, New Jersey). Pretac was applied to the disposable electrode surfaces (300–500 μL) before placement on the skin. Electrodes were positioned according to the standard bifrontal placement [22] with careful attention by avoiding the hairline to ensure uniform electrode-skin contact.

Skin impedance spectrum was measured using a FRA51615 frequency response analyzer (NF Corporation, Yokohama, Japan). Over three sequential trials, impedance spectrum measurements for each subject were carried out within the frequency range of 1–1000 Hz for 1 V, then 10–1000 Hz for 100 mV and then from 10 to 1000 Hz for 1 V. Before and after three impedance spectrum measurements, each subject's ‘static impedance’ was measured using the Thymatron System IV (Somatics LLC) ECT device.

The collected skin impedance spectrum data was decomposed to real and imaginary constituents of the 1–1000 Hz 1 V data set using EIS fitting software (EIS Spectrum Analyzer, (Bondarenko A. S., Ragoisha G. A. (2013) EIS Spectrum Analyser (1.0) Research Institute for Physical-Chemical Problems Belarusian State University) to fit an R-RC circuit model [23]. The EIS Spectrum analyzer software searched for best fit for values of components in the model so that the equivalent circuit represents the dataset using the Levenberg-Marquardt algorithm. After identifying the equivalent circuit (Rs, Rp and Cp) through the EIS Spectrum Analyzer for each subject, the impedance of these circuits was calculated for 1 kHz (NI Multisim 14.1, 1 kHz sinusoidal current source).

### 2.3. Experiment series 2: Influence of frequency or intensity on resulting impedance

A study to evaluate the influence of frequency or intensity on the resulting impedance using healthy subjects (n = 10) was conducted. For the purposes of this experiment, we applied the same frequency and pulse pattern that as used during dynamic impedance measurements (Fig. 2 B2) but at a low current intensity comparable to that used in static impedance measurement.

An arbitrary function generator (Tektronix AFG1022) was programmed to generate trains of rectangular, constant-current pulses with alternating polarity. The arbitrary function generator was connected to linear current isolator (Soterix Medical LCI) and the output of the current isolator was connected to the adherent electrodes (Thymapads, Somatics, LLC, Venice, FI). The skin was cleaned with saline and Pretac (Pharmaceutical Innovations, Newark, New Jersey) was applied. Electrodes were positioned according to the standard bifrontal placement, avoiding the hairline, with careful attention to ensure uniform electrode-skin contact. A biphasic waveform with amplitudes of 20 μA or 1 mA at 10 Hz with 3 different pulse widths (0.25 m s, 0.5 m s, 1 m s) was applied to each subject. Here, calculated impedance was the measured peak voltage divided by the applied peak current.

### 2.4. BF clinical ECT data set, imaging and segmentation

As described previously [18], anonymized data was re-analyzed from a North Shore- Long Island Jewish Health System ECT trial series [24] using bifrontal (BF) ECT. 4.2 × 4.9 cm disposable adhesive electrodes were used (Thymapads, Somatics LLC). Each subject received 6–10 ECT sessions with electrodes configured in a bifrontal montage with Pretac preparation. Static impedances were averaged across the first stimulation of each session, and dynamic impedances were averaged across the sessions where the seizure was generated for every stimulation. High resolution T1-weighted anatomical MRIs were deidentified from 4 subjects receiving ECT (subjects #21908, #22615, #22035, #21778). MR imaging exams were conducted at North Shore University Hospital on a 3T GE HDx scanner (General Electric, Milwaukee, WI, USA). Structural scans were acquired in the coronal plane using a three-dimensional spoiled gradient sequence (TR = 7.5 m s, TE = 3 m s, matrix = 256 × 256, FOV = 240 mm), producing 216 contiguous images (slice thickness = 1 mm) through the whole head.

**Fig. 1.**
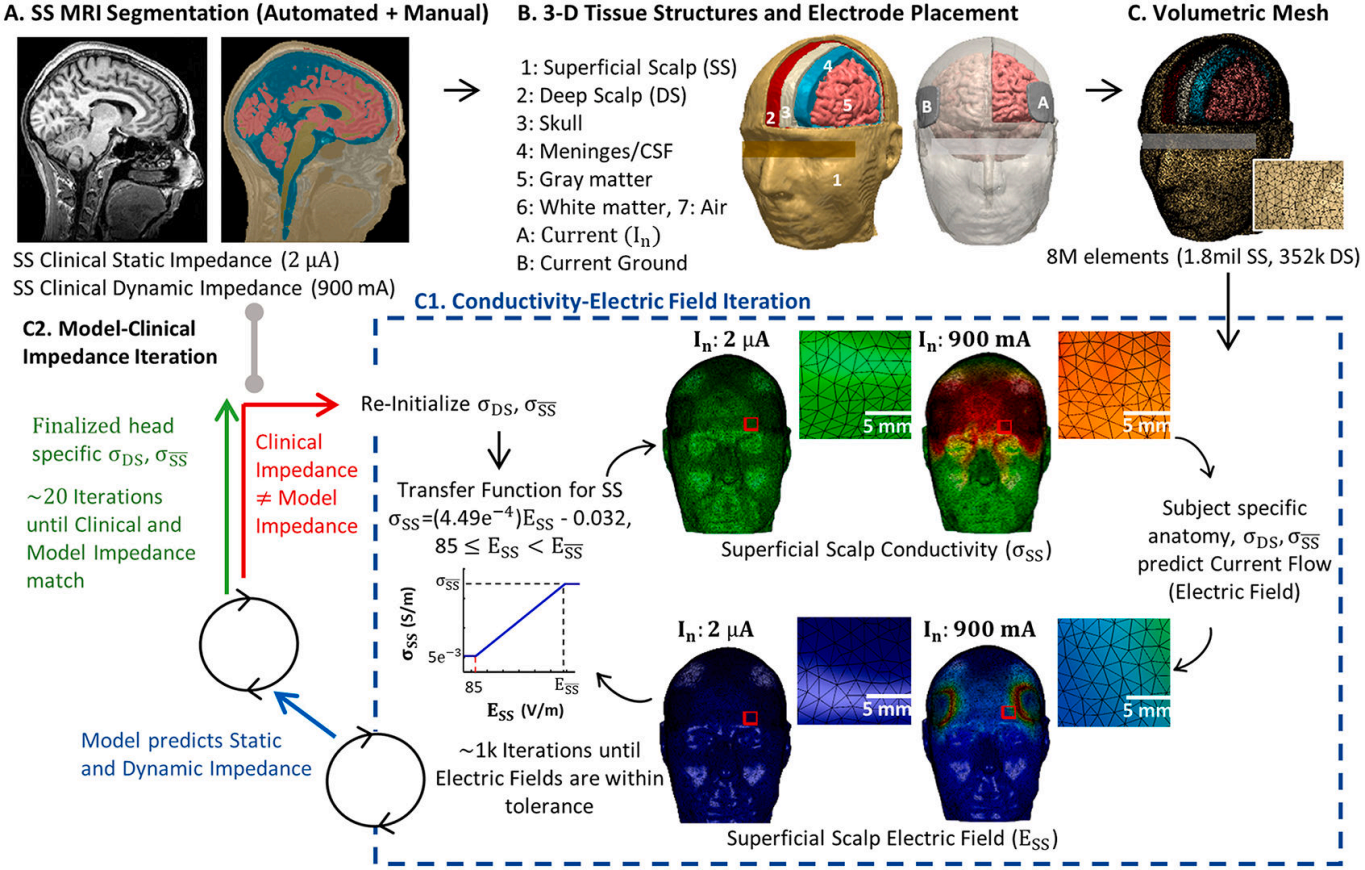
Revised adaptive computational pipeline for ECT current flow FEM models based at 1 kHz. In our previous study [17], we developed the first image-derived numerical models of transcranial electrical stimulation (tES) incorporating local changes in tissue conductivity in response to local current flow (electric fields). Here, we revised the transfer function based on our experimental results and to reflect the emphasis of our simulation at 1 kHz. (A) T1-weighted anatomical MRIs were collected from ECT patients with static impedance and dynamic impedance data. (B) Volume conductor models were created preserving image resolution using methods previously developed for low-intensity tES [8,26,27] - however, here, we divided scalp into Superficial Scalp (SS) and Deep Scalp (DS) compartments. Skull, meninges, gray matter, and white matter compartments were assigned standard fixed tissue conductivities. Clinical electrode montages were reproduced (e.g., BF) with boundary conditions corresponding to static impedance (I_n_ = 2 μA) and dynamic impedance (I_n_ = 900 mA) testing. (C) For each subject and electrode montage, a volumetric mesh was generated from the segmented data. (D) The model was initialized with a deep scalp conductivity ($\sigma_{\text{DS}}$) and a maximum superficial scalp conductivity ($\sigma_{\bar{\text{SS}}}$). Independently for 2 μA and 900 mA current, an iterative model then computed current flow based on tissue conductivities, updated superficial scalp conductivity ($\sigma_{\bar{\text{SS}}}$) in each element based on local electric fields using a transfer function, and then recalculated brain current flow (blue dashed square). The model converged after ~1 k iterations, producing a model prediction of static impedance (for 2 μA) and dynamic impedance (for 900 mA). (E) Model predicted static and dynamic impedance were compared with clinical static and dynamic impedance from the subject. If there was any significant mismatch, the model was reinitialized with updated deep scalp conductivity ($\sigma_{\text{DS}}$) and a maximum superficial scalp conductivity ($\sigma_{\bar{\text{SS}}}$), and the FEM was re-run until convergence (blue dashed square). When model static and dynamic impedance matched clinical data, a patient specific deep scalp conductivity ($\sigma_{\text{DS}}$) and a maximum superficial scalp conductivity ($\sigma_{\bar{\text{SS}}}$) was set.

Based on algorithms in SPM8 [25], updated for volume conduction models [26], an automated segmentation pipeline was used to create initial image masks of scalp, skull, air, meninges/cerebrospinal fluid, gray matter and white matter (Fig. 1 B). Additional manual segmentation was applied to correct for noise, aliasing artifacts, and to separate superficial scalp and deep scalp layers (approximately bisecting the scalp mask).

### 2.5. General modeling approach

We used the same selected subjects with the same general ‘adaptive’ FEM approach developed and described in our previous study [18] except with revised parameters (Table 1) of Equation (1) to reflect simulation at 1 kHz. Unless otherwise indicated, segmented tissues were assigned subject-independent and fixed (not electric field dependent) electrical conductivity [27]: skull (σ=0.01S∕m), gray matter (σ=0.276S∕m), white matter (σ=0.126S∕m), meninges/cerebrospinal fluid (σ=0.85S∕m), and air ($\sigma =1^{*}10^{-15}\;S∕m$). Deep-scalp layer was assigned a subject-specific but fixed (not electric field dependent) conductivity ($\sigma_{\text{DS}}$) between 4.5*10~^4^ S/m and 0.008 S/m. Local superficial-scalp layer conductivity ($\sigma_{\text{SS}}$) was a function of local scalp electric field ($E_{\text{SS}}$) as given by:

(Equation 1) σSS={A,0<ESS<BC∗ESS−D,B≤ESS<ESS¯σSS¯,ESS≥ESS¯}

Where $\sigma_{\bar{\text{SS}}}$ is subject-specific maximum superficial-scalp conductivity. $E_{\text{SS}}$ varies across the scalp surface such that $\sigma_{\text{SS}}$ then covaries across the superficial-scalp (e.g., higher near electrodes). Across subjects, four parameters (A, B, C, D) are fixed (Table 1). For each subject two model parameters were individualized: deep-scalp layer conductivity ($\sigma_{\text{DS}}$) and maximum superficial-layer conductivity $\sigma_{\bar{\text{SS}}}$. In addition, $E_{\bar{\text{SS}}}$ is subject specific as governed by Equation (1).

Stimulation electrodes were modeled in SolidWorks (Dassault Systèmes Corp., Waltham, MA). We represented ECT adhesive pad electrodes with dimensions of 4.2 × 4.9 cm and thickness of ~1.7 mm, and gel conductivity of 0.018 S/m. We modeled the bifrontal (BF) electrode placement as practically applied in the corresponding clinical series: the centers of both electrodes on the first imaginary line that originates at the lateral canthus and projects up parallel to the facial midline. The long edge of the electrodes aligned parallel to the first imaginary line such that the short edge of the pad electrodes is approximately parallel to the horizontal plane and right above supraorbital ridge (approximately above the eyebrow). Depending on the subject, the center of the electrodes is then ~3–5 cm above the canthus of the eye.

To summarize, the updated adaptive quasi-static-pipeline FEM ECT simulation of current flow is based on six assumptions.

1. Scalp is divided into two layers: deep-scalp and superficial-scalp.
2. Deep-scalp layer conductivity is isotropic, fixed and assigned a subject-specific value. This conductivity can be considered at 1 kHz or simply to be frequency independent (across a relevant range) so that it is applicable to all relevant frequencies, including the representative frequency of 1 kHz.
3. Local superficial-scalp layer conductivity increases instantly and linearly with local $E_{\text{SS}}$, starting at a threshold, up to a subject-specific maximum ($E_{\bar{\text{SS}}}$). At low-current, this conductivity represents 1 kHz, while at high intensities this conductivity represents ~0.01–2 kHz (so assuming conductivity does not vary in this range, which spans 1 kHz). At low-intensities, impedance is frequency dependent so 1 kHz is selected based on ECT device static impedance performance, while at high intensities, impedance is considered frequency independent over a relevant range (see Introduction).
4. Other tissues (skull, meninges/cerebrospinal fluid, gray/white matter, air) have subject-independent, fixed isotropic conductivities. These conductivities can be considered at ~1 kHz, or simply to be frequency independent (across a relevant range) so that it is applicable to all relevant frequencies, including the representative frequency of 1 kHz.
5. Electrode resistance is fixed and low (compared to body resistance) with a continuous interface with the skin.
6. Time is not explicitly considered (adaptive conductivity is instant). Capacitive effects/tissue permittivity are absent.

### 2.6. Computation and subject specific tissue parameterization

Computation was as previously described [18]. Modeled electrodes were incorporated into the segmentation. Volume meshes were generated from the segmented data and exported to COMSOL Multiphysics 5.5 (COMSOL Inc., MA, USA). The resulting mesh comprised >8,000,000 tetrahedral elements (>12,000,000° of freedom).

The Laplace equation ∇.(σ∇V)=0 (V: scalar electric potential; ∇: gradient vector; σ: conductivity) was solved and the boundary conditions were used such that current in static models (2 μ A) and dynamic models (900 mA; unless otherwise stated) is applied to one of the electrode terminals, while the other electrode is grounded. Superficial-scalp conductivity was expressed as a function of electric field (equation (1)). The finite element method (FEM) model was implemented using COMSOL. To converge the solution (Fig. 1C1), a linear system solver of conjugate gradients was used with a relative tolerance of 1*10^−3^ with a nonlinear system solver using the Newton-Raphson method (<500 iterations). This method is applied to millions of degrees of freedom iteratively.

An iterative approach (Fig. 1C2) was used to search for each subject-specific deep-scalp layer conductivity ($\sigma_{\text{DS}}$) and maximum superficial-layer conductivity ($\sigma_{\bar{\text{SS}}}$), such that model static impedance and dynamic impedance matched each subject's clinical static impedance and dynamic impedance values.

### 2.7. Statistical analysis

Normality of the Thymatron measurements, spectrum analyzer impedances at 1 kHz and equivalent circuit impedances at 1 kHz and the measured impedance of the same amplitude applied groups (20 μA and 1 mA) were assessed with Lilliefors test. Paired student's t-tests were performed between the static impedance measured before and after the three impedance spectrum measurements, and between the average Thymatron measurement and impedance at 1 kHz simulated from an equivalent circuit. Experimental data of subjects measured by the Impedance Analyzer at 1 kHz was analyzed using one-way (impedance analyzer trial index) ANOVA. Statistical significance and high correlation were considered when p < 0.05 and R^2^ > 0.7. A two-way ANOVA was performed to analyze the effect of amplitude and current pulse width effect on the impedance.

## 3. Results

### 3.1. Characterization of ECT device (Thymatron) output waveforms using resistor loads

Modern ECT devices deliver current-controlled pulses, so that the applied voltage is adjusted based on the encountered impedance. Devices report the resulting “dynamic impedance” during the passage of the ECT stimulus. Prior to stimulation, ECT devices report a “static impedance” using low intensity high frequency test currents. While static impedance and dynamic impedance has long been recognized as markers of individual differences and electrode setup, their etiology and consequences are poorly determined [28], including how they impact on seizure induction [29-32]. The current output waveform of ECT devices during therapy is relatively well characterized [33,34] whereas the (device specific) outputs used to probe static impedance are not. Moreover, how ECT machines calculate reported static impedances and dynamic impedances using the voltages needed to generate these currents should be understood, including for modeling of ECT.

**Fig. 2.**
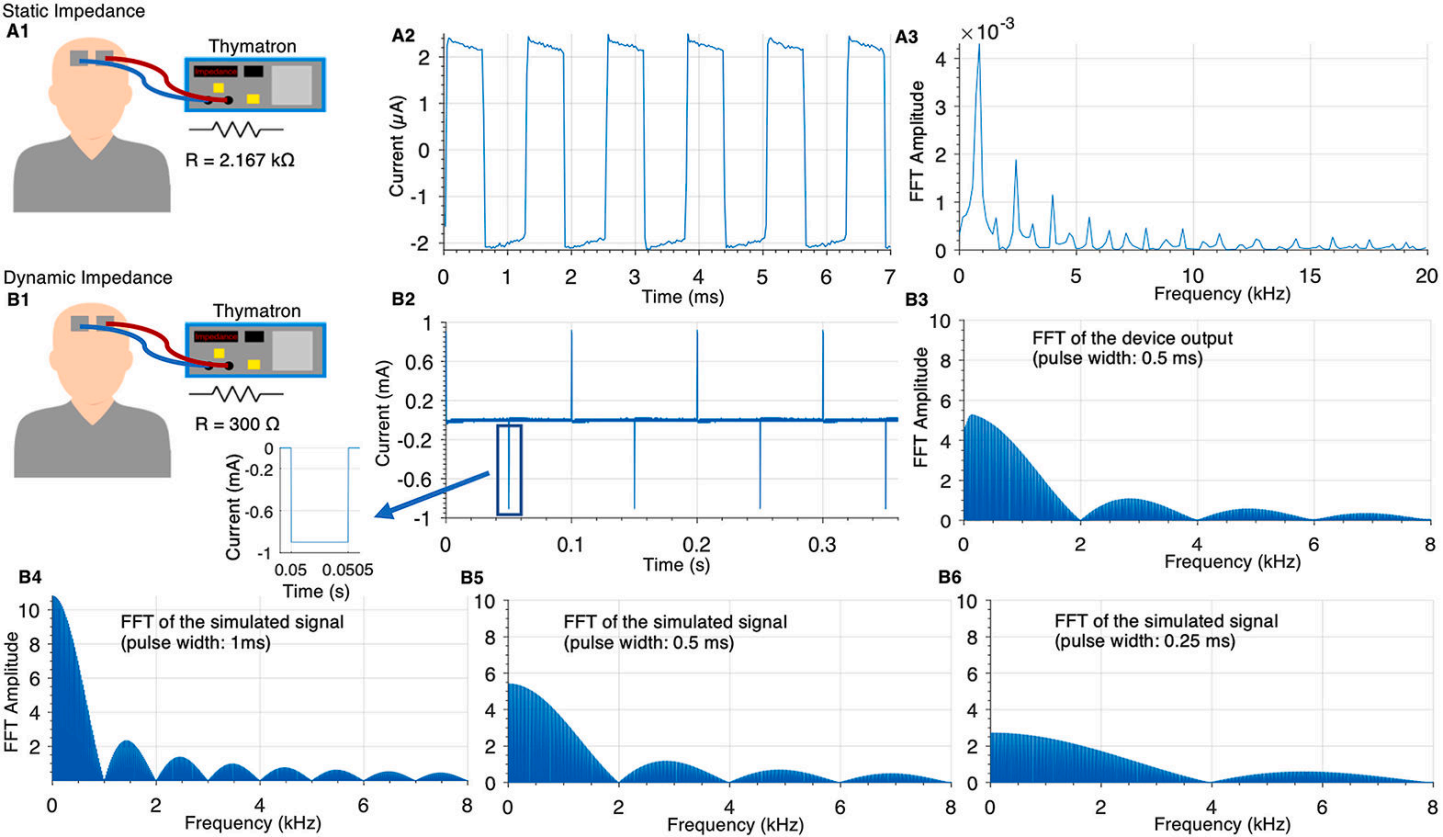
Thymatron device waveform during static and ECT (dynamic) impedance measurements across resistors. (A) Thymatron device static impedance measurement voltage across a 2.200 k Ω resistor that simulated human static impedance. The voltage divided by the resistance of the resistor gives the static impedance current waveform yielding the time-series waveform (~±2 μA peak square wave; A2) and its frequency content (A3). (B) Thymatron device dynamic impedance measurement voltage across a 300 Ω resistor to simulate human dynamic impedance. The voltage divided by the resistance of the resistor gives the dynamic impedance current yielding the time series (~±900 mA; B2) and its frequency content (B3). The frequency content of a simulated version of the idealized signal (Equation (2)) was calculated for different pulse widths (B4: 0.25 m s, B5: 0.5 m s; B6: 1 m s).

We measured the voltage output and analyzed frequency content of the Thymatron device waveform for each impedance case. The static impedance measurement voltage was measured across 2.200 kΩ resistor to approximate human static impedance. Using pure resistive loads, the Thymatron reported a static impedance close to the resistance of the applied load, in this case reporting 2.167 kΩ. The current waveform (Fig. 2 A2) generated (with a peak amplitude of ~2 μA) for measuring static impedance and its frequency content are shown (Fig. 2 A3). The waveform is approximately an 800 Hz biphasic square wave.

The voltage generated during ECT which is used to calculate dynamic impedance was measured across a 300 Ω resistor to approximate human dynamic impedance. Under pure resistive loads, the Thymatron reported a dynamic impedance that matched the applied load, in this case reporting 300 Ω. The current waveform (Fig. 2 B2) generated (with a peak amplitude of ~900 mA) during ECT and its frequency content are shown (Fig. 2 B3) under an ECT dose setting of 0.5 m s pulse width and 20 Hz. Note that pulse polarity alternates. The frequency content is consistent with an idealized alternating pulse-polarity waveform, as confirmed using a synthetic waveform with matched pulse width and frequency (Fig. 2 B5). The frequency content for a longer (1 m s; Fig. 2 B4) and shorter (0.25 m s; Fig. 2 B5) synthetic waveforms is also shown. Note that the first lobe of frequency content corresponding to the inverse of the pulse width (e.g., <2 kHz for 0.5 m s pulse width).

The function of simulated ECT output is a periodic signal $x_{T}(t)$ and is described at Equation (2). It is a pulse function with amplitude A and pulse width ∇ with a period of T. The frequency content can be described analytically by Equation (3) ∣Cn∣: The amplitude spectrum of the periodic signal $x_{T}(t)$, n: number of terms, A: amplitude T: period, Δ: pulse width). The analytical description matches and explains the experimental/numerically calculated results (Fig. 2) where the pulse repetition rate and pulse width set a lower and upper bound, respectively, for the main lobe frequency content.


(Equation 2) xT(t)={A,0<t<Δ−A,T2≤t<T2+Δ0,Otherwise}

(Equation 3) $$|Cn|=\frac{A}{\pi n}\;sin\left(\frac{\pi n\Delta }{T}\right)(1-{\left(-1\right)}^{n})$$

### 3.2. Experiments on healthy subjects using ECT electrodes 1: Static impedance and low-intensity impedance spectrum

In the first series of experiments on subjects, we aimed to characterize the frequency dependence of ECT electrode impedance (using an impedance analyzer), contrast these values with the static impedance reported by an ECT device, and consider if the frequency impedance spectrum is explained by a simple lumped parameter circuit model.

**Fig. 3.**
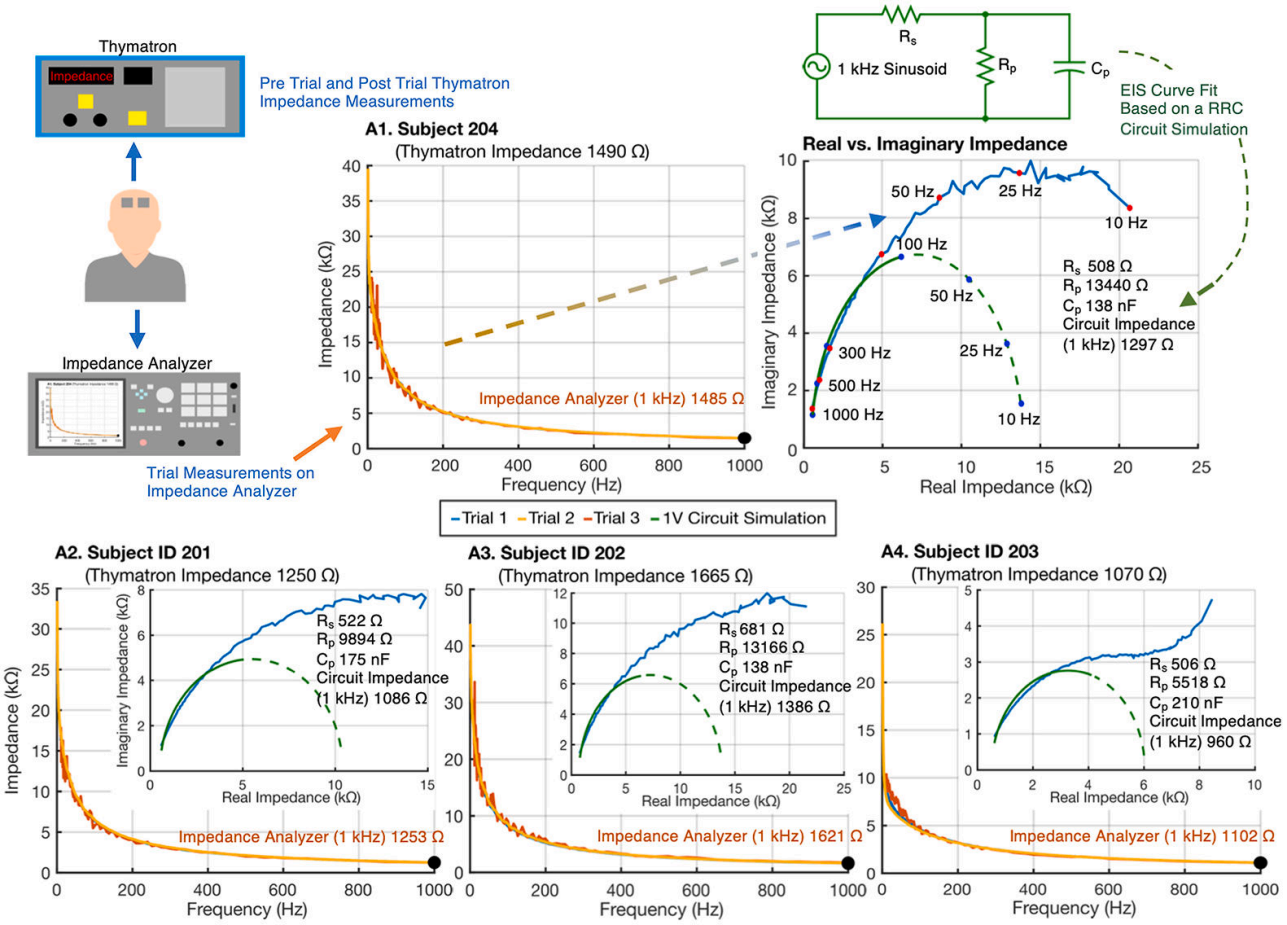
Analysis of low-current impedance and reported static impedance using ECT electrodes. The subject's static impedance was measured using a Thymatron System IV ECT device. Impedance was also measured using a frequency response (up to 1 kHz) analyzer device, with three repetitions (at 1 V, blue trace; 100 mV, orange trace; and then again at 1 V, yellow trace). For each subject, impedance decreased with frequency and at 1 kHz (orange text) approximated the Thymatron reported static impedance. The impedance frequency response was decomposed into real and imaginary constituents (blue traces). The impedance frequency response (only >100 Hz data) was used to parameterize a Rp, Rs, Cp circuit (green circuit) to approximate each subject's impedance data. For each subject, the best fit circuit parameter values are indicated, along with the frequency response of the circuit decomposed into real and imaginary constituents (green dashed trace) and the 1 kHz impedance of the circuit. While the Rp, Rs, Cp circuit impedance approaches a purely real value (of Rs + Rp) at low frequency, the experimental impedance real and imaginary components increase over the frequency ranges tested.

The impedance characteristic of ECT electrodes (Thymapads) under low currents was characterized on test subjects. An impedance spectrum analyzer was used alongside the Thymatron (Fig. 3, top left). Static impedance as reported by Thymatron device was compared with each subject's skin impedance as measured by an impedance analyzer device, including at 1 kHz (Fig. 3, orange, yellow traces). The real and imaginary components of the impedance analyzer data were also represented for each subject (Fig. 3, blue traces). Three repeated tests were taken by the impedance analyzer, preceding and followed by Thymatron static impedance measurements (Table 2).

Between repeated Thymatron measurements (before and after the three impedance spectrum measurements), there were no significant differences and a high correlation (p > 0.05, R^2^ = 0.94). There was no significant difference (F(_2, 27_) = 0.003, p > 0.05) among the repeated measures using impedance analyzer data at 1 kHz for each subject. Average Thymatron reported static impedance of subjects and the impedance analyzer 1 kHz data showed no difference and high correlation (p > 0.05, R^2^ = 0.99).

Impedance data from each subject (for >100 Hz frequency data only) was to fit a Rp, Rs, Cp circuit model (see Methods; Table 2). The impedance at 1 kHz of this circuit model was then calculated. The circuit-derived 1 kHz impedance and Impedance Analyzer measurement at 1 kHz was significantly different and highly correlated (p = 5.017e^−05^, R^2^ = 0.99, eta-squared = 0.85). Any Rp, Rs, Cp circuit is expected to have a purely real resistance of Rp + Rs at low frequency, and to have purely real resistance of Rs (Fig. 3, green traces) at high frequency. Whereas data collected by the impedance analyzer shows an increase in both real and imaginary impedance at low frequencies and so a deviation from a Rp, Rs, Cp circuit. Therefore, in fitting the aforementioned Rp, Rs, Cp circuit parameters for each subject, only data >100 Hz was considered.

We also considered if a single circuit Cp across subjects could reasonably model the impedance response across all subjects (at >100 Hz). However, we found that any single Cp value (e.g., the median of the subject-specific Cp values) results in significant changes in the predicted impedance at 1 kHz even changing the rank order across subjects (see Discussion for rationale and implication).

### 3.3. Experiments on healthy subjects using ECT electrodes 2: Influence of waveform resulting impedance

In the second series of experiments, we wanted to rule out the hypothesis that the difference between static impedance and dynamic impedance reflects difference in waveform (frequency content; Fig. 2) rather than differences in intensity. To address we measured impedance while applying stimulation with the waveform of ECT (as used during measurement of dynamic impedance) but low intensity (as used during measurement of static impedance).

In a sample of healthy subjects (n = 10), we measured impedance resulting from stimulation applied with alternating polarity pulse trains of varying pulse widths (0.25 m s, 0.5 m s, 1 m s) at 2 different peak amplitudes (20 μA and 1 mA). For 20 μA, the means of measured impedances for 0.25 m s, 0.5 m s, and 1 m s for 20 μA are, in order: 3932 ± 2287, 6400 ± 3980 and 10342 ± 6698. The means of measured impedances for 0.25 m s, 0.5 m s, and 1 m s for 1 mA are, in order: 3570 ± 1833, 5794 ± 3031, 9208 ± 5201. A two-way ANOVA revealed that there was not a statistically significant interaction between the effects of amplitude and current pulse width (F(2, 54) = 3.17, p > 0.05). Simple main effects analysis showed that amplitude did not have a statistically significant effect on impedance (p > 0.05) while current pulse width has a statistically significant effect on impedance (p = 0.0001, eta-squared = 0.39).

The subject's measured impedances were always in kiloohm range. However, when ECT is applied with a ~900 mA current, the impedance (i.e., dynamic impedance) is measured at being between 200 and 400 Ω range. This suggests the waveform (i.e., frequency and pulse pattern) applied during the ECT stimulation does not itself explain the drop in impedance, rather the current amplitude matters.

### 3.4. Development of updated individualized adaptive tissue models of ECT

We previously developed the first MRI-derived head models of tES incorporating current-dependent tissue properties and verified these models across ECT clinical data sets [18]. Since these models are adaptive, we can then simulate the tissue impedance changes from low-current (static impedance) to high-current (dynamic impedance) stimulation. This prior pipeline was (like other FEM pipelines) agnostic to frequency; here, we update the pipeline to center on 1 kHz under the rationale that this tracks ECT device reported static impedance and dynamic impedance (see Introduction).

The analysis was applied to data from four ECT patients' identifying maximum superficial scalp conductivity ($\sigma_{\bar{\text{SS}}}$) and the deep scalp conductivity ($\sigma_{\text{DS}}$) that produced predicted static and dynamic impedance values corresponding to the patient's clinical data. The resulting subject specific parameters were: Subject 21908, $\sigma_{\bar{\text{SS}}}=0.16\;S∕m$ at $E_{\bar{\text{SS}}}\geq 428\;V∕m$, $\sigma_{\text{DS}}=0.002\;S∕m$; Subject 22035, $\sigma_{\bar{\text{SS}}}=0.5\;S∕m$ at $E_{\bar{\text{SS}}}\geq 1185\;V∕m$, $\sigma_{\text{DS}}=4.5^{*}10^{-4}\;S∕m$; Subject 22035, $\sigma_{\bar{\text{SS}}}=0.3\;S∕m$ at $E_{\bar{\text{SS}}}\geq 740\;V∕m$, $\sigma_{\text{DS}}=0.008\;S∕m$; Subject 21778, $\sigma_{\bar{\text{SS}}}=0.4\;S∕m$ at $E_{\bar{\text{SS}}}\geq 963\;V∕m$, $\sigma_{\text{DS}}=0.0012\;S∕m$.

**Fig. 4.**
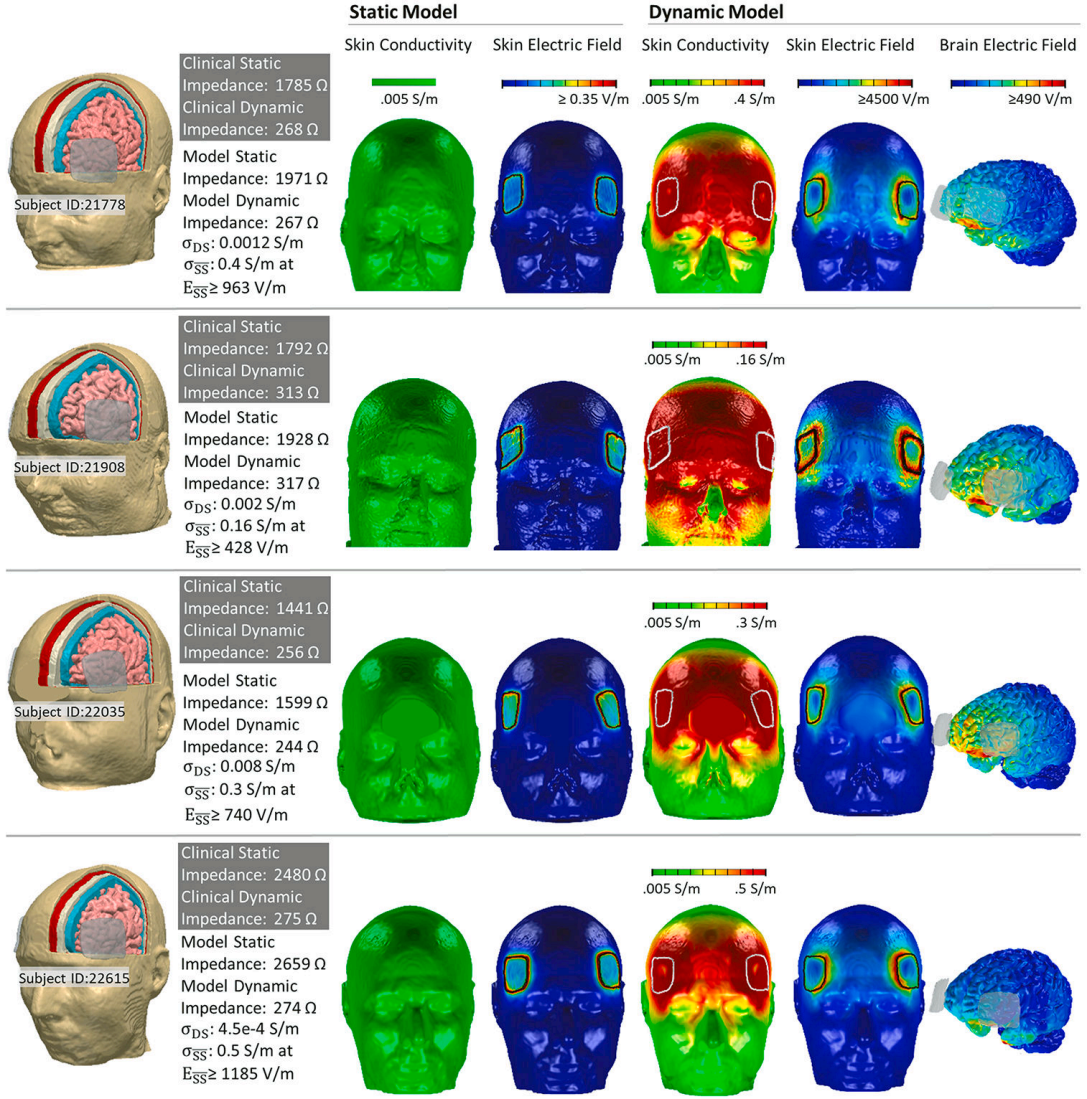
Adaptive (1 kHz based) ECT models for four ECT subjects. Dynamic FEM models simulated current flow across four subjects who have received ECT (Subject IDs: 21778, 21908.22035, 22615). (First Column) Model anatomy was based on subject anatomical MRI. Static impedance and dynamic impedance values were recorded for each subject (gray box). Each subject model was assigned a specific deep scalp conductivity ($\sigma_{\text{DS}}$) and a maximum superficial scalp conductivity ($\sigma_{\bar{\text{SS}}}$) as indicated, such that adaptive FEM simulation predicted corresponding static impedance (based on 2 μA applied current) and dynamic impedance (based on 900 mA applied current) as indicated. (Second and Third Column) Results from the static impedance (2 μA current) simulation showed resulting superficial scalp conductivity and scalp electric field. (Third, Fourth, Fifth Column) Results from the dynamic impedance (900 mA current) simulation showed resulting superficial scalp conductivity, scalp electric field, and brain electric field. We emphasize in these novel adaptive simulations that brain current flow was determined by tissue conductivity and superficial scalp conductivity was simultaneously determined by local electric field. Even for the 2 μA (static) model local changes in scalp conductivity are predicted. For the 900 mA (dynamic) model, the saturation of the transfer function between superficial scalp electric field and conductivity resulted in a more diffuse saturation of scalp conductivity (front of head) compared to scalp electric field (around electrodes).

Stimulation with 2 μA (Fig. 4, static model) produced peak scalp electric fields under and around electrode edges (<0.85 V/m) with no increase in conductivity around the electrode perimeters. Simulations with 1 mA applied current, using this updated pipeline, produces a predicted static impedance of 1822 Ω for Subject 21908, 2443 Ω for Subject 22615, 1865 Ω for Subject 21778, 1522 Ω for Subject 22035 (not shown); these values are 4.8–8.1% less for 2 μA. Stimulation with 900 mA (Fig. 4, dynamic model) produced high electric fields across the scalp forehead with peaks around electrodes (>4500 V/m) and an associated increase in scalp conductivity (0.15–0.5 S/m). The predicted brain current flow during ECT results in peak electric fields >490 V/m. A detailed view of superficial skin current and conductivity during ECT is represented for Subject 22615 (Supplementary Fig. 1).

## 4. Discussion

How is the complex nature of tissue impedance during ECT reconciled with the use of a quasi-static assumption (without frequency-dependent tissue conductivity)? The approach developed here, as explained in the Introduction, is based on a quasi-static pipeline using tissue conductivity only at 1 kHz. In ECT modeling, our approach should either be made explicit (whether conventional or adaptive ECT models) or an alternative rationalized.

### 4.1. Measurements supporting 1 kHz based adaptive modeling pipeline for ECT

Notwithstanding a potential decrease in ECT electrode impedance over minutes [18], we collected data with sufficient expediency to minimize time effects (Table 2). We found no significant difference in impedance spectrum between 1 V and 100 mV tests (Table 2) or 20 μA and 1 mA, consistent with these test current intensities below those starting to produce meaningful current-dependent impedance changes. These intensifies are still above those generated by the Thymatron static impedance circuit with 2 μA, corresponding to ~5 mV. Repeated measure with the impedance analyzer and Thymatron static impedance provide assurance that the test currents were not directly producing lasting skin changes. Thus low-current measurements correspond to conditions where electric fields in the skin are not sufficient to produce meaningful changes in conductivity – as reflected in the updated adaptive ECT modeling pipeline (Equation (1), Fig. 4 static model).

While, *a priori*, one might expect the Thymatron to report an impedance reflecting 800 Hz test current frequency (Fig. 2 A2), we found the Thymatron reported a static impedance close to the 1 kHz value determined by the impedance analyzer. This can easily be explained by idiosyncrasies of the Thymatron circuit/processing, and also serves to emphasize that static impedance is a device-specific measurement [18]. The Thymatron reliably reports as ‘static impedance’ the resistance of a resistor, but does not reliably report the 1 kHz impedance of any arbitrary circuit (i.e., any RC circuit). The coincidence of Thymatron reported static impedance values with 1 kHz impedance is specific to the electrodes and conditions tested. Nonetheless, this supports our proposition that modeling head impedance at 1 kHz will predict reported Thymatron static impedance. The pattern of current flow through the brain at low-current or impedance at other frequencies are not essential questions.

The ECT waveform consists of alternating phase pulses (Fig. 2 B2) [33-37]. Pulses have a broad frequency content, but for 0.25–1 m s pulse width, most of the power is 100 Hz (first harmonic) to 4 kHz (first lobe at 0.25 m s). We cannot repeat impedance analyzer measurements for ECT-level currents (~900 mA). If impedance varied significantly with frequency (e.g., there was significant capacitance component to impedance) then ECT voltage output during therapy will be distorted compared to an ideal square wave; but prior recordings suggest a square voltage output [34]. Further unpublished data (James Long, personal communication, using Mecta device) shows 1) minimum reactive component during ECT; 2) dynamic impedance matches resistance as calculated by peak pulse voltage divided by applied current; and 3) resistance changes <13% across sequential ECT pulses. Taken together, these support our proposition that impedance is largely resistive during ECT (within 100 Hz-4 kHz), so that tissue conductivity can be considered frequency invariant under high currents. This, in turn, justifies modeling the ECT at a representative frequency (e.g., 1 kHz). Specifically, in our pipeline a 1 kHz conductivity is fixed for deep tissues, and 1 kHz conductivity increases with local electric field for superficial skin (Equation (1)).

### 4.2. When and where frequency matters in transcranial electrical stimulation

Typical tES models are frequency agnostic, ignoring the role of frequency or assuming it is irrelevant. This is implicit in low-intensity direct current [9], low-intensity AC [38-41], and prior ECT models [35,42,43] including our initial development of adaptive ECT models [18]. Whereas models of tES conventionally focus on brain electric fields, our concerns also include device-reported impedances that may be especially sensitive to skin conditions. Moreover, for ECT we address broad frequency content waveforms (Fig. 2 A3, B3) that span very low (to 2 μA) to very high (800–900 mA) intensity, so amplifying concerned about frequency and intensity-dependent tissue conductivity.

The impact of current intensity and frequency on skin impedance is not in doubt [17,44], including the use or extra-low (tens of uA) current to measure “impedance” before tES [45]. For 0.25–1 mA intensities and 0.1–150 Hz tES, experiments suggest tissue can be approximated as fixed conductivity [19,46-48]. Still, measurements of intra-cranial electric fields show an incremental impact of frequency, with ~11% reduction in electric field from 1 Hz to ~100 Hz [19,46,49], consistent with increasing skin conductivity. When intensity is increased from 2 to 4 mA, moderate increases in skin conductivity can explain reductions in impedance with small reduction in brain electric fields [50]. The importance in tES on complex tissue conductivity may depend on cases where higher model precision is required and/or applications using higher intensity/frequency waveforms (e.g., temporal interference stimulation; [51-53]).

The sources of impedance include the electrode-interface (boundary between the conductor and hydrogel electrolyte), remainder of electrode assembly [54] components, the electrode-skin interface, skin, and deep tissues. The contribution of the electrode-assembly components and electrode-interface can be shown to be negligible by measuring two electrodes directly connected (~50 Ω irrespective of intensity). The skin-electrode interface is not an electrode interface, but rather a continuous boundary between two electrolyte conductors (as is the interface between tissues). So, one cannot attribute to the skin-electrode interface properties of electrodes [55]. Could the skin-electrode interface account for the intensity and frequency (Fig. 3) dependent impedance? Electrode design [56-58] and (ECT) preparation [18] will impact impedance in so far as they govern current flow through the skin-electrode boundary. But we are not aware of data attributing any (significant) complex impedance to a “skin-electrode interface” (except fully dry electrodes that are not suitable for stimulation). Rather, decades of skin impedance measurement have shown frequency and intensity dependence *inside* specific skin layers and micro-structure [59,60]; i.e., layers that do not interface with the electrode.

### 4.3. Lumped parameter models on skin impedance

Copious literature models complex skin impedance with lumped parameter circuits [61-67]. Lumped parameter circuits can represent complex impedance in a few skin segments, but cannot account for spatial dispersion at the skin or deeper (brain) current flow patterns. We first developed adaptive FEM models of current flow [18] to account for current-dependent tissue conductivity, which were frequency agnostic. To model frequency, as an alternative approach to the single-frequency FEM described above, we considered using FEM to predict total resistance (e.g., at DC) and then integrate this prediction into a lumped parameter model with additional capacitive elements, to predict frequency-sensitivity. After substantial effort, this FEM plus lumped-parameter approach was not successful but did yield insights on the nature of electrode/body impedance.

Lumped parameter models of skin impedance have varied complexity [64,68]. We considered the most basic R_s_, R_p_, C_p_ circuit (Fig. 3, top right). Assuming the FEM models predict DC impedance, then Rs and Rp might be derived from the subject-specific FEM model, but C_p_ cannot. However, if a subject-independent value of C_p_ can be applied, the resulting lumped parameter model can predict frequency-specific impedance. Ultimately, the intensity dependence of Rs or Rp could also be modeled. However, while experimental impedance could be approximated with this circuit (at frequencies >100 Hz; Fig. 3 green trace), this was only reliable with subject-specific C_p_ values (Table 2).

The circular arc of real vs imaginary impedance (Fig. 3, green lines) is known to apply to skin [59]; the deviation we observed at frequencies <100 Hz (Fig. 3, blue lines) may reflect the electrode-skin interface. To the extent static impedance is intended to predict dynamic impedance, and if this deviation is not relevant to impedance during ECT, it is rational ECT devices report static impedance approximating >100 Hz (e.g., 1 kHz) impedance.

### 4.4. Biophysical basis and the quasi-static pipeline

If one does not ignore complex impedance, then without the 1 kHz simplification developed here or the ability to adapt lumped parameter models, another alternative is to model the underlying biophysics. For tES, this is computationally intractable and indefinitely parameterized when one considers the skin is composed of multiple layers, each with distinct non-linear impedance (both resistance and capacitance are not linear; [17,62,65]). Moreover, microscopic appendages such a sweat glands and blood-vessels transverse skin layers. Even assuming fixed conductivity, these layers and appendages are themselves complex to model in a small patch a skin [20,69], and for tES the entire scalp would need to be represented.

This biophysical approach introduces a level of microscopic nuance (e.g., voltages changes over μm) that is not relevant for explanations on the scale of phenomena detectable and impactful to tES (e.g., voltage changes over mm, overall head impedance). Our goal was to adapt quasi-static tES FEM modeling for ECT, accounting for intensity- and frequency-dependent tissue conductivity. Our simplifying assumptions are explicit (see Methods). This approach remains state-of-the-art and better than simply ignoring the underlying issues.

### 4.5. Conclusion: How to model ECT

We summarize five approaches to FEM (anatomically precise) modeling current flow during ECT.

1. Under the quasi-static assumption with fixed resistivity, which needs to be reconciled with intensity dependent (as evidenced in difference in static vs dynamic impedance, and impacting individual difference [18]) and frequency dependent impedance (shown here).
2. Hybrid quasi-static FEM with a hybrid lumped-parameter model [5], which cannot represent regional tissue impedance or account (in our approach) for individual differences.
3. A biophysical model, which demands extensive micro-scopic parametrization and is computational intractable except for small skin patches [20,69].
4. Solving the inhomogeneous electromagnetic wave (Maxwell's) equations across all tissues [1,4] with varied degrees of simplification (e.g., no propagation or induction). The simplifications of impedance insensitivity to intensity and to frequency (which allows the quasi-static assumption) are, as explained here, unjustified for ECT.
5. The approach developed and experimentally validated here, using a quasi-static pipeline at a representative (device and waveform specific) frequency, which further allows subject-specific models referencing static and dynamic impedance. This approach should not be confused with invoking the quasi-static assumption, but it does rescue prior ECT models to be rationalized under a quasi-static pipeline.

## Supplementary Material

Figure S1

## Figures and Tables

**Table 1:** Parameters and corresponding values represented in Equation (1).

Parameters	Values
A	5*10–3
B	85
C	4.49*10–4
D	0.032

**Table 2:** Subject impedance measurements at low currents with ECT electrodes. Table includes Thymatron (pre, post and average measurements) measurements, impedance analyzer measurements at 1 kHz, and the lumped circuit values approximating impedance spectrum (>100 Hz data only) with the 1 kHz impedance of that circuit. EIS curve fit element parameters are for Trial 1. Note, impedance analyzer Trial 1 is for 1 V (10–1000Hz), Trial 2 is 1 V (1–1000Hz), and 100 mV (10–1000 Hz). IMP: Impedance.

Subject ID	Thymatron IMP (Ω)			IMP Analyzer 1 kHz (Ω)			Circuit Values			IMP 1 kHz(Ω)
	Pre	Post	Avg.	Trial 1	Trial 2	Trial 3	R_s_ (Ω)	R_p_ (kΩ)	C_p_ (nF)	
201	1210	1290	1250	1253	1255	1242	522	9.89	175	1086
202	1690	1640	1665	1621	1630	1690	681	13.17	138	1386
203	1000	1140	1070	1102	1111	1089	506	5.52	210	960
204	1460	1520	1490	1485	1490	1467	508	13.44	138	1297
205	1670	1710	1690	1673	1716	1690	555	22.59	119	1475
206	1090	1090	1090	1033	1055	1040	449	4.24	219	906
207	850	770	810	808	808	804	372	2.84	294	810
208	1370	1370	1389	1389	1357	1376	557	6.70	160	1203
209	1020	900	939	939	904	980	432	2.57	263	806
210	940	830	885	838	818	862	374	3.97	277	723
