# Supplementary figures and images for: Quasi-static pipeline in electroconvulsive therapy computational modeling
Source: Brain Stimul. Author manuscript; Available in PMC 2024 Apr 3. (PMC10988926; doi:10.1016/j.brs.2023.03.007)

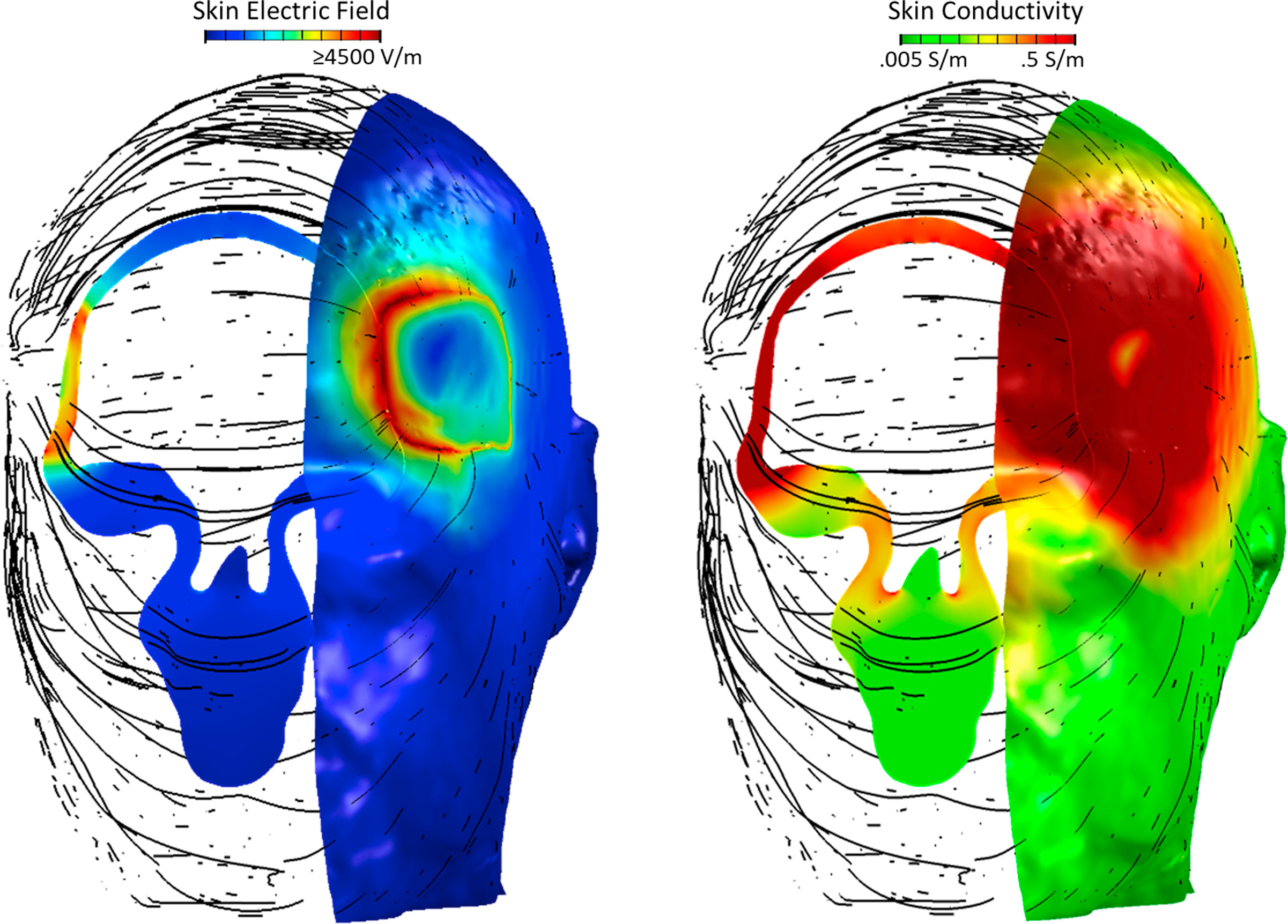

Supplement: Figure S1 [file NIHMS1977653-supplement-Figure_S1.jpg]
